# Macronutrient and carbon supply, uptake and cycling across the Antarctic Peninsula shelf during summer

**DOI:** 10.1098/rsta.2017.0168

**Published:** 2018-05-14

**Authors:** Sian F. Henley, Elizabeth M. Jones, Hugh J. Venables, Michael P. Meredith, Yvonne L. Firing, Ribanna Dittrich, Sabrina Heiser, Jacqueline Stefels, Julie Dougans

**Affiliations:** 1 School of GeoSciences, University of Edinburgh, James Hutton Road, Edinburgh EH9 3FE, UK; 2 University of Groningen, PO Box 11103, 9700 CC Groningen, The Netherlands; 3 British Antarctic Survey, High Cross, Madingley Road, Cambridge CB3 0ET, UK; 4 National Oceanography Centre, European Way, Southampton SO14 3ZH, UK; 5 Scottish Universities Environmental Research Centre, Rankine Avenue, East Kilbride G75 0QF, UK

**Keywords:** nutrients, carbon cycling, nitrate isotopes, nitrogen cycle, Antarctic Peninsula, Circumpolar Deep Water

## Abstract

The West Antarctic Peninsula shelf is a region of high seasonal primary production which supports a large and productive food web, where macronutrients and inorganic carbon are sourced primarily from intrusions of warm saline Circumpolar Deep Water. We examined the cross-shelf modification of this water mass during mid-summer 2015 to understand the supply of nutrients and carbon to the productive surface ocean, and their subsequent uptake and cycling. We show that nitrate, phosphate, silicic acid and inorganic carbon are progressively enriched in subsurface waters across the shelf, contrary to cross-shelf reductions in heat, salinity and density. We use nutrient stoichiometric and isotopic approaches to invoke remineralization of organic matter, including nitrification below the euphotic surface layer, and dissolution of biogenic silica in deeper waters and potentially shelf sediment porewaters, as the primary drivers of cross-shelf enrichments. Regenerated nitrate and phosphate account for a significant proportion of the total pools of these nutrients in the upper ocean, with implications for the seasonal carbon sink. Understanding nutrient and carbon dynamics in this region now will inform predictions of future biogeochemical changes in the context of substantial variability and ongoing changes in the physical environment.

This article is part of the theme issue ‘The marine system of the West Antarctic Peninsula: status and strategy for progress in a region of rapid change’.

## 1. Introduction

Southern Ocean biogeochemical processes play a critical role in the redistribution of nutrients and other chemical species between the major ocean basins, in air–sea CO_2_ exchange, and consequently in modulating global climate over seasonal, interannual and millennial time scales [1–4]. The Antarctic continental shelves are particularly important for the biological uptake of CO_2_ due to higher area-normalized primary production rates than any other Southern Ocean region [5].

The West Antarctic Peninsula (WAP) continental shelf is one such region of high primary productivity, supporting a large and productive food web [6,7]. Primary production is paced by the annual sea ice cycle, being negligible over winter and maximal during summer, when large phytoplankton blooms can develop under favourable upper ocean conditions where demands for light, iron and macronutrients are met [8,9]. The timing and magnitude of phytoplankton blooms is regulated by the extent and duration of ice cover and its effect on upper ocean stability, thus the light conditions to which phytoplankton are exposed [10,11]. Increased or longer-duration sea ice cover leads to higher primary production, by sheltering the upper ocean from wind-driven mixing during winter and spring, resulting in a shallow well-lit mixed layer favourable for phytoplankton growth during summer [12–14]. Because changes in primary production have strong consequences for higher trophic levels, this sea ice-driven variability can influence the functioning of the entire ecosystem [15,16].

The primary source of macronutrients and dissolved inorganic carbon (DIC) to the WAP shelf system is warm, nutrient- and carbon-rich Circumpolar Deep Water (CDW), which intrudes onto the shelf from the Antarctic Circumpolar Current, and persists there year-round below approximately 200 m [17–21]. Shelf-break processes, mesoscale eddies and deep glacially scoured canyons are particularly important for the cross-shelf transport of CDW [22–24]. Marguerite Trough is one of the largest canyons acting as a conduit for CDW from the shelf break to the inner shelf, and is thus a major part of the flow at depth in the central WAP [25]. As CDW crosses the shelf, it is modified by mixing with overlying Antarctic Surface Water (AASW) [26]. Mixing is strongest during winter, due to intense winds, surface cooling and brine rejection during sea ice formation [27–29], and this brings nutrients and CO_2_ from CDW to surface waters [30–33]. Meltwater inputs and solar radiation during summer freshen and warm the surface waters, restratifying the upper ocean and isolating the remnant Winter Water as a temperature minimum (*T*_min_; <−1°C) layer between AASW and CDW [34]. This *T*_min_ layer carries the biogeochemical signatures of surface waters from the previous winter, but can be modified by mixing with water masses above and below, and by subsurface nutrient remineralization.

Nutrient and carbon supply by deep winter mixing and drawdown by phytoplankton utilization during summer drives a strong seasonal cycle in mixed layer concentrations, which are highest during winter and decrease to mid-summer, before replenishment by renewed mixing into autumn and remineralization of organic matter as the phytoplankton bloom subsides [6,30,33]. WAP phytoplankton communities consist of diatoms, as well as cryptophytes, mixed flagellates, prasinophytes and haptophytes [14,35,36], such that silicic acid drawdown and recycling is an important part of the regional biogeochemistry [33,37,38]. While macronutrients are mostly replete over the WAP shelf, short-lived nutrient limitation has been documented in coastal regions when drawdown is intense [30], with nutrient supply by wintertime mixing suggested as the limiting factor of availability [39]. Sea ice-driven interannual variability in primary production is imprinted on seasonal nutrient drawdown; high-ice years with stable upper ocean conditions and large phytoplankton blooms lead to greater nutrient drawdown than in low-ice low-productivity years [30,39].

In most years, primary production creates a seasonal biological sink for CO_2_ [40,41]. Estimates of organic matter export over the WAP shelf vary in time, space and between different methodologies, but up to approximately 50% of surface primary production can be removed to depth, with both particle sinking and passive transport of particulate and dissolved organic matter playing important roles [42–45]. In addition to water mass mixing, primary production and export, seawater carbonate chemistry along the WAP is regulated by sea ice processes, glacial meltwater and organic matter respiration and remineralization [31,46–48]. High seasonality and spatial variability in upper ocean carbon dynamics have been observed over the WAP shelf [32,47,49], and decadal enrichment in inorganic carbon and acidification have been documented to the north [50].

Remineralization of organic matter and regeneration of nutrients and CO_2_ in the high-latitude Southern Ocean is most intense following the high-productivity summer period, with nitrification of ammonium to nitrate occurring in the mixed layer during autumn and winter when light levels are low [51–53]. Mixed layer nitrification has also been observed during spring and summer in the deep mixed layers around the Kerguelen Plateau [54,55]. In the WAP region, organic matter remineralization and nitrification have been shown to have a significant impact on upper ocean nutrient biogeochemistry in coastal areas, with potential consequences for larger-scale biogeochemical cycles [30].

In addition to its ecological and biogeochemical importance, the WAP is notable for pronounced atmospheric and oceanic warming, sea ice losses and widespread glacial retreat during the latter part of the twentieth century [56–60]. Increases in the heat content and prevalence of CDW over the shelf have been identified as an important factor driving these changes [24,60], and may influence the supply of macronutrients and carbon to shelf ecosystems, with potential feedbacks on air–sea gas exchange and ocean–climate interactions [19].

### (a) Objectives

The primary objective of this study was to examine the processes influencing the delivery of macronutrients and DIC from CDW to the surface ocean across the WAP shelf. Venables *et al.* [61] showed that CDW loses heat, salt and density from the mouth of Marguerite Bay along the deep channel, which is thought to be the dominant flow path of CDW, into Ryder Bay. This loss is driven by localized mixing events associated with topographic overflows of shallower waters as the deepest, densest waters are progressively blocked by multiple transverse ridges along the channel. This study aims to examine the biogeochemical modification of CDW occurring in parallel with these physical processes, and elucidate the driving mechanisms and consequences for primary production and nutrient drawdown.

The seasonal dynamics of macronutrients and inorganic carbon have been studied in detail in Ryder Bay alongside the Rothera Time Series (RaTS) program [30,32,37,49]. A secondary objective of this study was to gain a regional perspective on the key processes at work in this coastal location, in particular the regeneration of nutrients and carbon through organic matter remineralization and nitrification [30]. Here we examine these processes across the shelf and their contribution to regional nutrient budgets and cycling. A large number of studies have focused on nitrate isotope systematics in the Southern Ocean [51,53–55,62–68]. This is the first study to examine nitrate isotopes across the Antarctic continental shelf with such high sampling resolution to understand the key nitrogen cycle processes at work. Understanding nutrient and carbon supply, uptake and cycling at the WAP now will help us to develop predictive skill regarding how these processes may respond and feed back to ongoing changes in the coupled ice–ocean–atmosphere system.

## 2. Material and methods

**Figure 1.**
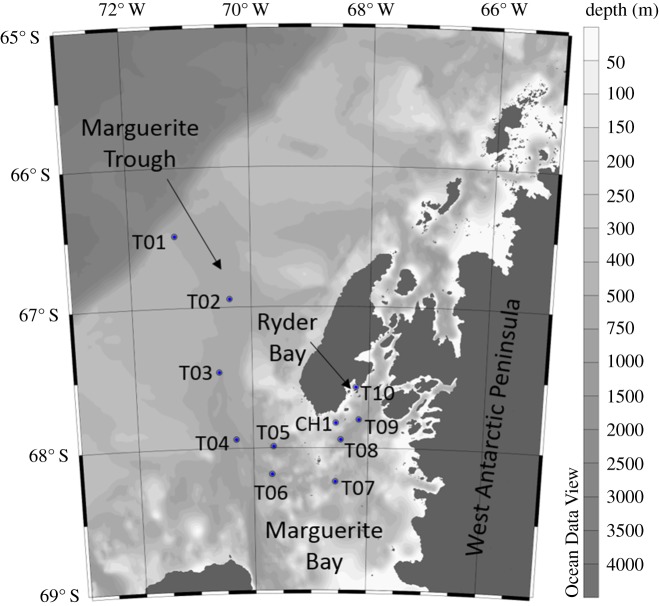
Map of the study area showing all 11 stations and the locations of Marguerite Trough, Marguerite Bay, Ryder Bay and the WAP mainland. Grey shading depicts bathymetry, according to the colour bar shown. (Online version in colour.)

This study was conducted on cruise JR307 aboard the British Antarctic Survey's RRS James Clark Ross in January 2015. Eleven stations were sampled along a transect across the WAP shelf, from the mouth of the glacially carved canyon Marguerite Trough, along this trough into Marguerite Bay and into Ryder Bay (figure 1). With the exception of station CH1, the stations lie along the path of the CDW water mass from the shelf break to Ryder Bay, where the RaTS program is conducted. Station CH1 (cold hole 1) is the location of a bathymetric depression where the temperature below 200 m is markedly cooler than over most of the shelf, due to its topographic isolation from warm CDW [61].

At each station, a full-depth conductivity–temperature–depth (CTD) cast was taken with a Seabird SBE911Plus package, comprising dual SBE3Plus temperature and SBE4 conductivity sensors and a Paroscientific pressure sensor. Photosynthetically active radiation (PAR) and fluorescence were also measured by the CTD package using a LICOR PAR sensor and a Chelsea AquaTracka 3 fluorometer. The CTD conductivity sensors were calibrated using samples taken at depth and within the mixed layer and analysed for salinity (a function of conductivity and temperature) using the onboard Guildline 8400B Autosal salinometer.

A RDI Workhorse lowered acoustic Doppler current profiler (LADCP) attached to the CTD rosette measured velocity in 8 m vertical bins. A value for the vertical eddy diffusivity (*K_z_*) in the top 300 m of each profile is estimated from LADCP velocity profiles and CTD stratification using an internal wave-based parametrization, following Kunze *et al.* [69]. Finestructure parametrizations of turbulence are subject to considerable uncertainty [70], but nevertheless are useful for assessing patterns of spatial and temporal variability. The estimates produced here, of 10^−6^ to 10^−5^ m^2^ s^−1^, are towards the lower end of previous finestructure shear estimates in the region [29,71], while estimates either based on bulk parametrizations [24,72,73] or from direct microstructure turbulence observations (M. Inall 2017, personal communication) are even larger.

Water samples were taken over the full water column depth at each station on the upcast of CTD deployments from 12 litre Niskin bottles mounted on the 24-bottle rosette. Bottles were sampled for carbonate system parameters, immediately after the Niskin was opened, then macronutrients, isotopic composition of nitrate, particulate organic carbon (POC), particulate nitrogen (PN), oxygen isotopes of seawater and salinity for CTD calibration.

Samples for carbonate chemistry were drawn into 500 ml borosilicate glass bottles, preserved with 100 μl saturated mercuric chloride solution and stored for analysis. Analyses for DIC and total alkalinity were conducted at Rothera Research Station using a VINDTA 3C (Marianda) following the method of Jones *et al.* [49]. The precision of DIC and alkalinity measurements was ±1.7 and ± 1.5 µmol kg^−1^, respectively, based on the average difference between in-bottle duplicate analyses of Certified Reference Material batch 130 (*n *= 54). Seawater partial pressure of CO_2_ (*p*CO_2_) and pH on the total seawater scale were calculated from DIC and alkalinity, with *in situ* temperature, salinity, pressure and macronutrient concentrations using the CO2SYS program [49].

Samples for macronutrient and nitrate isotope analysis were filtered using Acrodisc PF syringe filters with 0.2 µm Supor membranes, snap frozen at −80°C for 12 h and then stored at −20°C for subsequent analysis in the UK. Prior to nutrient analysis, samples were thawed for 48 h to ensure complete redissolution of secondary silicate precipitates to silicic acid. Concentrations of nitrate + nitrite, nitrite, phosphate and silicic acid were analysed using a Technicon AAII segmented flow autoanalysis system with reference materials from General Environmental Technos Co. (Japan) at Plymouth Marine Laboratory, UK. Raw data were corrected to elemental standards and ambient ocean salinity and pH. Samples were assayed in duplicate and standard deviation was generally better than 0.2 µmol l^−1^ for nitrate + nitrite, 0.01 µmol l^−1^ for nitrite, 0.02 µmol l^−1^ for phosphate and 0.6 µmol l^−1^ for silicic acid. Nitrate concentration was obtained by differencing nitrate + nitrite and nitrite measurements.

Analysis of the stable isotope composition of nitrogen (*δ*^15^N) and oxygen (*δ*^18^O) in nitrate was performed at the University of Edinburgh using the bacterial denitrifier method and gas chromatography isotope ratio mass spectrometry (GC-IRMS) [74–77]. Briefly, denitrifying bacteria (*Pseudomonas aureofaciens*) were grown on agar plates and in tryptic soy broth (30 g l^−1^ milli-Q water) amended with sodium nitrate (1 g l^−1^), ammonium sulfate (0.25 g l^−1^) and potassium phosphate monobasic (5 g l^−1^), and used for the quantitative conversion of sample nitrate to N_2_O gas. Bacteria were isolated from tryptic soy broth by centrifugation after 6–8 days and resuspended in nitrate-free media, 3 ml aliquots of which were purged with N_2_ gas for 3 h before being injected with seawater sample volumes to provide 20 nmol of nitrate. After denitrification overnight, NaOH was added to samples to lyse bacterial cells and scavenge CO_2_. Sample N_2_O was analysed using a Thermo Finnigan DeltaPlus Advantage mass spectrometer with a CTC Analytics GC Pal autosampler and a Thermo Finnigan Gas Bench II gas preparation system. Results are presented in the delta per mille (‰) notation relative to international standards, atmospheric N_2_ for N (*δ*^15^N‰_AIR_) and Vienna Standard Mean Ocean Water (VSMOW) for O (*δ*^18^O‰_VSMOW_), after raw sample data were referenced to IAEA-NO3 and USGS-34 standards. Analytical precision was around ±0.2‰ for N and around ±0.3‰ for O. The *δ*^18^O value was corrected for fractionation during conversion of nitrate to N_2_O, for exchange with seawater oxygen during denitrification, and for blanks using the correction scheme of the Sigman Laboratory, Princeton University [77].

Nitrite was not removed prior to denitrification. Although the contribution of nitrite to the nitrate + nitrite pool is low throughout this study (less than 3%), nitrite *δ*^15^N has been shown to be extremely low in the Southern Ocean [64]. Initially, our data are presented as measured as *δ*^15^N and *δ*^18^O of nitrate + nitrite (
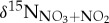
 and 
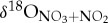
), with *δ*^18^O corrected for the differential O loss during denitrification between nitrate and nitrite, following the correction scheme of Kemeny *et al.* [64]. In the discussion, 
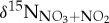
 values are used to estimate 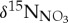 by correcting for nitrite interference using measured concentrations of nitrite and nitrate + nitrite and published values for 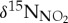 of −24 ± 38‰ for samples ≥100 m depth and −69 ± 33‰ for samples <100 m [64]. These corrections are similar to those detailed in Henley *et al.* [30], except that the range of 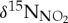 values used here for upper ocean samples is significantly lower than in the previous study (−24‰). As a result of this approach, we are careful to distinguish between 
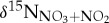
 and 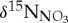 throughout. The 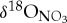 data are taken to be equal to 
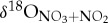
, as *δ*^18^O of nitrate + nitrite has been shown to be very similar to that of nitrate-only analyses [64].

Samples for POC and PN analysis were filtered through muffle-furnaced 25 mm GF/F filters (nominal pore size 0.7 µm) using a custom-built overpressure system. Filters were dried overnight, snap frozen at −80°C and stored at −20°C for analysis at the University of Edinburgh. Similar to Henley *et al.* [78], samples were decarbonated prior to analysis by rewetting with milli-Q water and fuming with 50% HCl for 24 h and then dried at 50°C overnight. Samples were analysed for POC and PN concentration and the isotopic composition of PN (*δ*^15^N_PN_) using a Carlo Erba NA 2500 elemental analyser in-line with a VG Prism III IRMS. The *δ*^15^N_PN_ data were referenced to atmospheric N_2_ (‰_AIR_) using PACS isotopic reference material. POC and PN concentrations were calibrated to an acetanilide elemental standard. Analytical precision was around ±0.2‰.

Samples for determination of the ratio of stable oxygen isotopes of seawater (*δ*^18^O) were taken directly from the Niskin into 50 ml glass bottles, which were immediately sealed with stoppers and crimp caps. These were stored in the dark at +4°C during transport to the UK, where they were analysed at the British Geological Survey, Keyworth. Samples were analysed using the equilibrium method for oxygen [79], with samples run on a VG Isoprep 18 and SIRA 10 mass spectrometer. The *δ*^18^O data were standardized relative to VSMOW and duplicate analyses indicated an average precision better than ±0.02‰. The *δ*^18^O and salinity data are used here in a simple three-endmember mass balance that quantifies separately the contributions to the freshwater budget of each sample from sea ice melt and meteoric water (the sum of glacial discharge and direct precipitation). This was developed originally for the Arctic [80], and was used most recently at the WAP by Meredith *et al.* [81], which presents full details on the procedure.

## 3. Results

### (a) Cross-shelf trends in physical, macronutrient and carbonate system parameters

**Figure 2.**
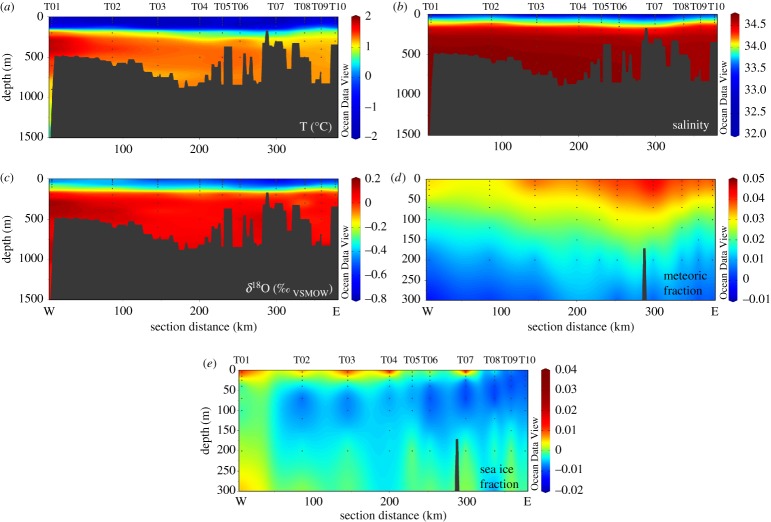
Section plots of (*a*) temperature, (*b*) salinity and (*c*) *δ*^18^O and derived fractions of (*d*) meteoric water and (*e*) sea ice meltwater along the transect from T01–T10. CH1 is excluded due to its topographic isolation from CDW.

Physical oceanographic data (figure 2) show the characteristic water column structure for the WAP shelf during summer. Maximum temperatures (above 1°C) associated with modified CDW (mCDW) were observed below 200 m across the shelf (figure 2*a*). This mCDW was overlain by the *T*_min_ layer between approximately 100 and 25 m, with temperatures below −1°C marking the Winter Water mass. The uppermost AASW water mass varies in temperature up to 0.5°C. Salinity is highest at depth in the mCDW (34.61 ± 0.12) and decreases towards the surface to a minimum of 32.01 at station T07 (figure 2*b*).

Across-shelf trends show loss of heat, salt and density as CDW flows along Marguerite Trough and the deep channel into Ryder Bay, with greater modification after stations T05 and T06 where there are more sills [61]. The *δ*^18^O value is low in the surface ocean compared to CDW and decreases across the shelf (figure 2*c*), with lowest values near shore reflecting the combined influence of glacial discharge and orographic effects on precipitation [81]; this is also reflected in the derived meteoric water fraction (figure 2*d*). The sea ice contribution is largest in surface waters, particularly at T01, T03, T04 and T07 (figure 2*e*), the latter being the only station within the southward-retreating ice pack at the time of sampling. Negative values in the *T*_min_ layer at all but the furthest offshore station reflect net sea ice formation during the preceding winter.

**Figure 3.**
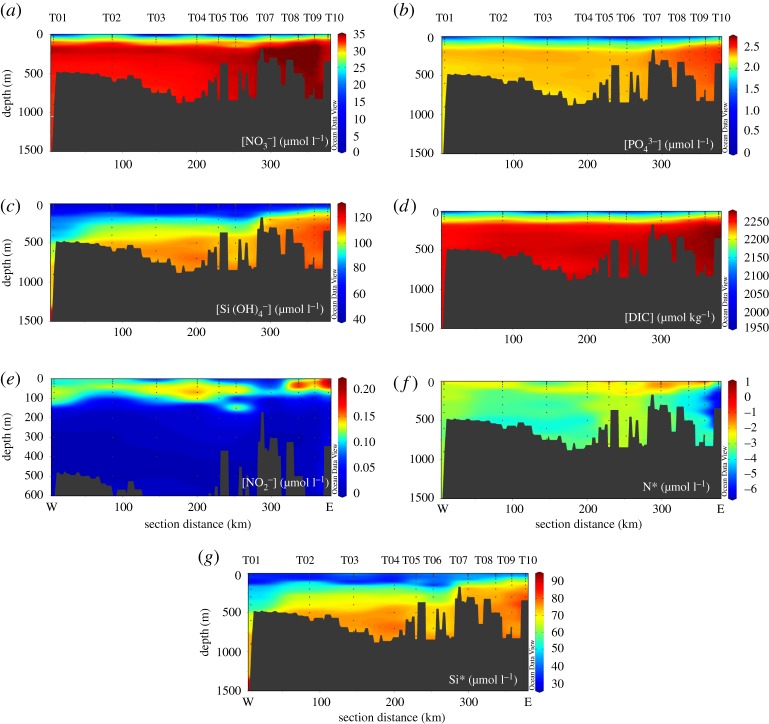
Section plots of concentrations of (*a*) nitrate, (*b*) phosphate, (*c*) silicic acid, (*d*) DIC and (*e*) nitrite and (*f*) N* and (*g*) Si* along the transect as for figure 2.

Nitrate, phosphate and silicic acid are enriched at depth and nutriclines shoal with distance across the shelf, opposite to the trends indicated by physical parameters (figure 3*a–c*). Nitrate and phosphate enrichments are strongest at 100–300 m depth, while silicic acid increases continuously to the bottom and the strongest cross-shelf increases occur in the deepest waters. Nitrate is highest at T08–T09, phosphate is highest at T10 and silicic acid is highest at T08–T10. DIC also increases below the mixed layer across the shelf, with maximum values at T09–T10 (figure 3*d*). Nitrite concentration is highest in the shallow subsurface and increases in general across the shelf, with maxima at T08 and T10 (figure 3*e*).

N* ([NO_3_^−^] − 16[PO_4_^3−^]; [82]) and Si* ([Si(OH)_4_^−^] − [NO_3_^−^]; [83]) are used here to describe the deviations of nitrate from phosphate concentrations and of silicic acid from nitrate, respectively. Ammonium was not measured, so is not included in these calculations. N* decreases with depth, with the largest decreases at the innermost stations, T09, T10 and CH1 (figure 3*f*). N* at depth decreases inshore from T05 and T06, reaching significantly lower values at CH1 and T10 than all other stations (*p* = 2.17 × 10^−13^, two-sample *t*-test). Cross-shelf changes in N* at depths ≥100 m are negatively correlated with phosphate (*r*^2^ = 0.631, *p* = 2.24 × 10^−16^, *n* = 70), rather than nitrate. Si* increases with depth, following silicic acid, and is highest in the deepest waters at most stations, with maxima in inner-shelf regions (figure 3*g*).

### (b) Concentrations and isotopic signatures of nitrogen and carbon with depth

**Figure 4.**
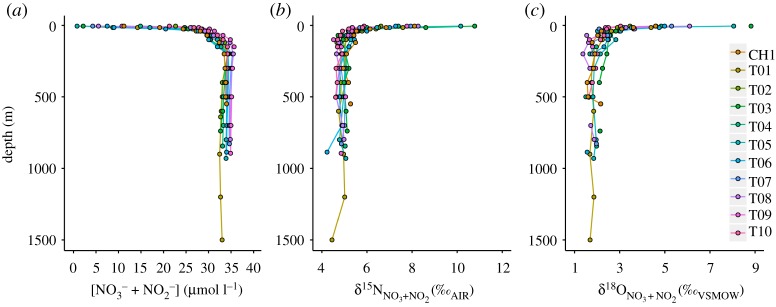
Depth profile plots of (*a*) [NO_3_^−^ + NO_2_^−^], (*b*) 
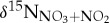
 and (*c*) 
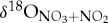
 for all stations, as per legend.

Nitrate concentration in CDW (≥200 m depth) is 34.0 ± 0.8 µmol l^−1^ (figure 4*a*). Inner-shelf stations show maximum concentrations up to 35.68 µmol l^−1^ at 100–200 m. All stations show nitrate drawdown towards the surface, to values as low as 0.76 µmol l^−1^ at station T05. The value of 
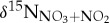
 in CDW is 4.9 ± 0.2‰ (figure 4*b*) and 
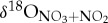
 is 1.9 ± 0.3‰ (figure 4*c*), in agreement with literature values [62,64,66]. Both increase into the surface ocean as nitrate is taken up by phytoplankton, up to values of 10.8‰ for 
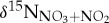
 and 8.8‰ for 
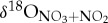
.

**Figure 5.**
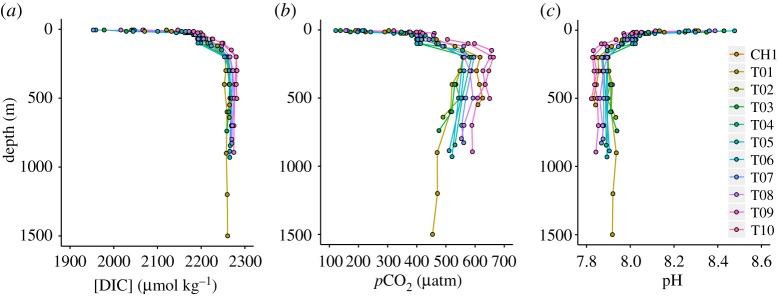
Depth profile plots of (*a*) DIC concentration, (*b*) *p*CO_2_ and (*c*) pH for all stations, as per legend.

DIC concentration is highest in mCDW with values of 2267 ± 8 µmol kg^−1^ and varies across the shelf from 2257 ± 3 µmol kg^−1^ at the outer shelf (T01) to 2281 ± 1 µmol kg^−1^ in Ryder Bay (T10) (figure 5*a*), in good agreement with literature values [47,49]. DIC decreases in the surface ocean to values as low as 1953 µmol kg^−1^. The *p*CO_2_ value in mCDW is 567 ± 50 µatm and shows a clear increase along the transect from 506 ± 42 µatm at T01 to 649 ± 11 µatm at T10 (figure 5*b*). *p*CO_2_ increases upwards at each station to a maximum at 200 m, before decreasing into the surface ocean to values as low as 122 µatm. pH is 7.88 ± 0.03 in CDW and shows the opposite trend to *p*CO_2_ (figure 5*c*). *p*CO_2_ maxima and pH minima at 200 m occur as a result of high DIC relative to alkalinity (not shown), which lowers buffering capacity, while low *p*CO_2_ and higher pH in the surface layer are driven by biological drawdown lowering DIC relative to alkalinity, thus increasing buffering capacity.

**Figure 6.**
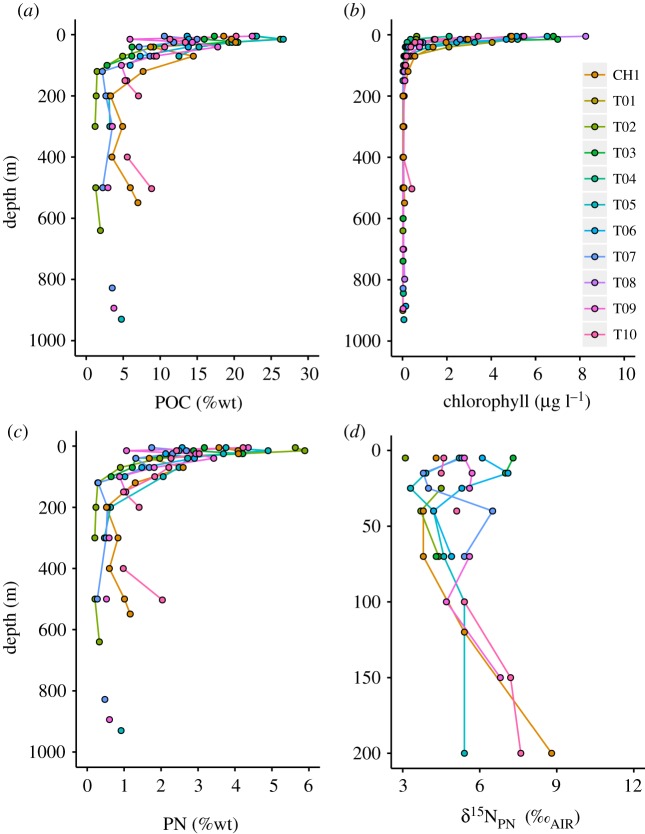
Depth profile plots of (*a*) weight percent POC, (*b*) chlorophyll, (*c*) weight percent PN and (*d*) *δ*^15^N_PN_ for all stations, as per legend, which applies to all plots. Note different *y*-axis scale for *d*.

The weight percent organic carbon (%OC) and nitrogen (%N) in suspended particulate matter are maximal in the surface ocean, up to 26.7%OC and 5.9%N, and decay rapidly over the upper 200 m to generally less than 7.5%OC and less than 1.5%N, as organic matter sinking out of the surface ocean is remineralized (figure 6*a,c*). *δ*^15^N_PN_ varies between 3.1‰ and 7.3‰ in the upper ocean, and increases to values as high as 8.8‰ below the euphotic surface layer, in concert with substantial decreases in %N (figure 6*d*).

### (c) Chlorophyll and macronutrient fluxes and deficits

Chlorophyll concentration derived from CTD fluorescence is highest in the upper 15 m and decreases rapidly to less than 0.25 µg l^−1^ below 70 m (figure 6*b*). Surface chlorophyll is patchy along the transect, with highest values at T03, T05 and T08. The depth interval with chlorophyll greater than 0.5 µg l^−1^ extends deepest below the mixed layer at T01, T05, T06 and CH1, suggesting higher export at these stations.

Chlorophyll integrated over the upper 100 m varies from 31 to 231 mg m^−2^ and is greatest at T05, followed by T01, T03, T09 and CH1 (table 1). Nitrate deficit varies from 51 to 378 mmol m^−2^ and is greatest at T03, T05 and T06. DIC deficit varies from 321 to 2857 mmol m^−2^ and is also greatest at T03, T05 and T06. Silicic acid deficit varies from 28 to 318 mmol m^−2^ and is greatest at T07, followed by T01, T05 and T06.

**Table 1.** Chlorophyll integrated over the upper 100 m, fluxes of nitrate, silicic acid and DIC into the upper ocean, *K_z_* used to calculate fluxes, deficits of nitrate, silicic acid and DIC, and the uptake ratios of [Si(OH)_4_^−^]/[NO_3_^−^] and [NO_3_^−^]/[PO_4_^3−^] from linear regressions of macronutrient concentrations (figure 7*a,b*). Uptake ratios are only given for statistically significant relationships (*p* < 0.05); NS denotes no significant relationship. Uncertainties are standard errors. Macronutrient deficits are computed relative to wintertime values, assuming that the high concentrations at 40 m (25 m for T09 and T10) measured during the cruise are found all the way to the surface in winter. Macronutrient fluxes were calculated by multiplying the nutrient–depth gradient between the uppermost sample of mCDW and the surface sample by estimated *K_z_*.

	chlorophyll (100 m) (mg m^−2^)	*K*_z_ (10^−5 ^m^2^ s^−1^)	nitrate flux (mmol m^−2^ d^−1^)	nitrate deficit (mmol m^−2^)	Si flux (mmol m^−2^ d^−1^)	Si deficit (mmol m^−2^)	DIC flux (mmol m^−2^ d^−1^)	DIC deficit (mmol m^−2^)	[Si(OH)_4_^−^]/[NO_3_^−^]	[NO_3_^−^]/[PO_4_^3−^]
T01	183.90	0.71	0.13	204.55	0.12	273.27	0.67	2144	NS	15.3 ± 0.3
T02	30.85	0.39	0.03	106.76	0.09	67.62	0.24	1036	NS	14.1 ± 0.4
T03	178.24	0.64	0.16	376.45	0.08	197.92	0.80	2857	0.6 ± 0.1	15.7 ± 0.3
T04	49.62	2.24	0.24	129.91	0.45	28.13	2.15	935	NS	14.8 ± 0.4
T05	231.37	0.75	0.21	322.16	0.26	278.63	0.99	2557	0.8 +0.04	15.3 ± 0.1
T06	153.06	3.83	0.56	377.71	0.49	271.99	2.44	2641	0.8 ± 0.1	15.7 ± 0.4
T07	159.35	1.90	0.41	172.85	0.67	318.09	2.63	1469	1.1 ± 0.2	15.3 ± 0.4
T08	97.37	0.18	0.04	157.94	0.07	257.05	0.20	1051	1.2 ± 0.1	14.7 ± 0.3
T09	182.04	0.26	0.06	121.11	0.13	190.77	0.25	825	1.4 ± 0.2	13.4 ± 0.2
T10	95.15	0.36	0.04	51.22	0.12	85.08	0.21	321	1.5 ± 0.1	12.4 ± 0.4
CH1	191.02	0.33	0.05	222.20	0.09	262.56	0.28	1570	1.0 ± 0.1	13.0 ± 0.4

*K_z_* varies substantially along the transect, with lowest values less than 4 × 10^−6^ m^2^ s^−1^ at the northern Marguerite Bay stations (T08–T10, CH1) and on the outer shelf (T02), and the highest value close to 4 × 10^−5^ m^2^ s^−1^ at the mouth of Marguerite Bay (T06) (table 1). Macronutrient fluxes for each station were calculated by multiplying the nutrient–depth gradient between the uppermost sample of mCDW and the surface sample by the estimated value for *K_z_*. As such, variability in both nitrate and silicic acid fluxes is driven principally by *K_z_* (nitrate: *r*^2^ = 0.881, *p* = 1.9 × 10^−5^; silicic acid: *r*^2^ = 0.667, *p* = 0.0022). Nitrate flux shows the same pattern as *K_z_* and varies from ≤0.1 to 0.56 mmol m^−2^ d^−1^. While nitrate and silicic acid fluxes are positively correlated along the transect, with a slope of 1.0 ± 0.2 (*r*^2^ = 0.757, *p* = 0.0005), silicic acid flux is largest in central Marguerite Bay (T07) and smallest at T02, T03, T08 and CH1. DIC fluxes follow a similar pattern to *K_z_* and nitrate, except that they are highest in central Marguerite Bay. Our nitrate fluxes are smaller than previously reported for the WAP shelf [73] as a result of our lower estimates of *K_z_*.

**Figure 7.**
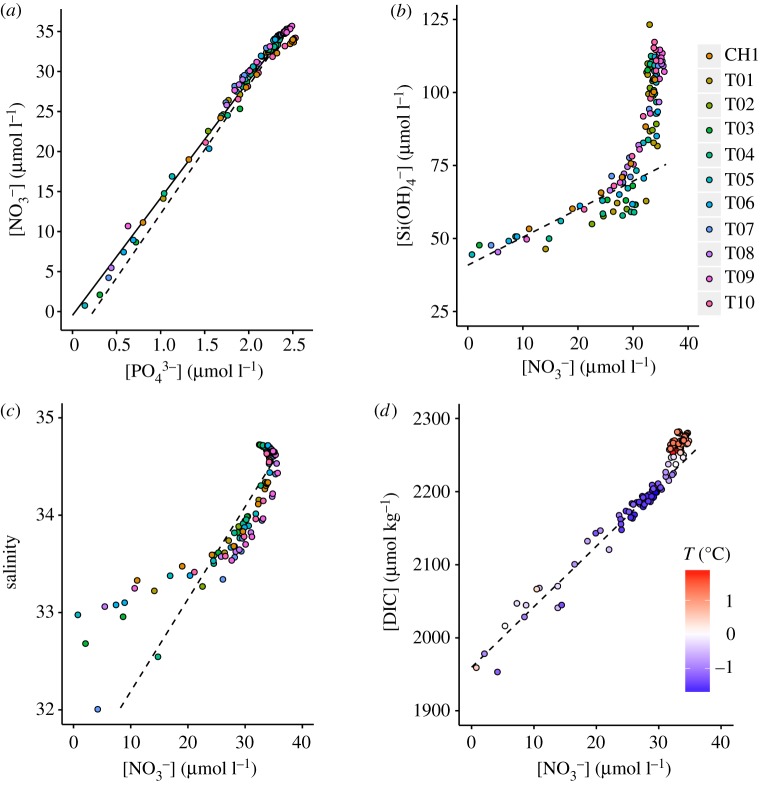
Plots of (*a*) nitrate versus phosphate, (*b*) silicic acid versus nitrate, (*c*) salinity versus nitrate and (*d*) DIC versus nitrate for all stations. Note different colour shading and legends; stations for plots (*a–c*) as per the legend next to (*b*), temperature for (*d*). In (*a*), the dashed line depicts uptake according to the Redfield ratio (16 : 1); the solid line is the linear regression for our data with a [NO_3_^−^]/[PO_4_^3−^] uptake ratio of 14.6 ± 0.2. In (*b*), the dashed line depicts the linear regression for the upper 40 m where biological uptake occurs, with a [Si(OH)_4_^−^]/[NO_3_^−^] uptake ratio of 1.0 ± 0.1. In (*c*), the dashed line shows a mixing trend between the high-nitrate high-salinity subsurface waters and the upper ocean. In (*d*), the dashed line shows the linear regression from the surface to the Winter Water layer.

Nutrient uptake ratios estimated from the slopes of linear regressions of nutrient concentrations (table 1) show that the [NO_3_^−^]/[PO_4_^3−^] uptake ratio over the whole study is 14.6 ± 0.2 (s.e., *n* = 119, *r*^2^ = 0.983, *p* < 2.2 × 10^−16^; figure 7*a*). The plot of [Si(OH)_4_^−^] versus [NO_3_^−^] (figure 7*b*) shows a piecewise-linear relationship, with a [Si(OH)_4_^−^]/[NO_3_^−^] uptake ratio of 1.0 ± 0.1 (s.e., *n* = 40, *r*^2^ = 0.755, *p* = 1.7 × 10^−13^) at depths ≤40 m. These values indicate that, overall, [NO_3_^−^]/[PO_4_^3−^] uptake ratios were lower than the Redfield ratio [16N : 1P] and [Si(OH)_4_^−^]/[NO_3_^−^] showed the expected 1 : 1 ratio for diatom-dominated primary production. Some important variability exists across the shelf, with [NO_3_^−^]/[PO_4_^3−^] uptake consistent with the Redfield ratio at stations T01, T03, T05, T06 and T07, lower values at T02, T04 and T08, and the lowest values at T09, T10 and CH1. [Si(OH)_4_^−^]/[NO_3_^−^] uptake in the upper 40 m is < 1 : 1 at stations T03, T05 and T06, and ≥1 : 1 at T07, T08, T09, T10 and CH1. Figures 7*c* and 7*d* show relationships of nitrate with salinity and DIC, and are discussed in §§4a and 4f.

## 4. Discussion

### (a) Supply and uptake of nutrients across the shelf

Nitrate, phosphate, silicic acid, DIC and *p*CO_2_ in subsurface waters all increase across the shelf to maxima in inner-shelf regions (figure 3). In the context of CDW being a source of heat, salt, nutrients and CO_2_ to the WAP shelf, these enrichments are contrary to the reduction in heat, salinity and density of subsurface waters as CDW crosses the WAP shelf, as a result of blocking of the deeper, less modified CDW and topographic overflows of shallower waters [61]. The relationship between salinity and nitrate over the full water column depth (figure 7*c*) shows a mixing trend between the highest-concentration subsurface waters and the fresher surface waters; deviations to the left of the mixing line at lower concentrations and salinities show the influence of biological uptake in the surface ocean. Similar relationships with salinity exist for phosphate and DIC, while silicic acid shows a more linear relationship with a smaller deviation (i.e. less drawdown) in the surface samples. Despite the mixing signatures visible in each vertical profile, increasing subsurface nutrient concentrations across the shelf do not lead to a parallel increase of parametrized nutrient fluxes from mCDW to the surface ocean (table 1).

Biological variables are correlated in the upper ocean, with statistically significant (95% confidence level) positive relationships found between 100 m integrated chlorophyll and nitrate deficit (*r*^2^ = 0.366, *p* = 0.048), DIC deficit (*r*^2^ = 0.394, *p* = 0.039) and silicic acid deficit (*r*^2^ = 0.604, *p* = 0.0049). In addition to the strong positive correlation between nitrate and DIC deficits (*r*^2^ = 0.927, *p* = 2.0 × 10^−6^), this indicates nutrient and carbon uptake during primary production, with close coupling of carbon and nitrogen, and the importance of diatoms to the phytoplankton community overall. However, the lack of a direct relationship between nitrate and silicic acid deficits (*p* = 0.08) reflects a varying diatom contribution across the shelf. Mixed layer depth does not show a strong relationship with chlorophyll (*p* = 0.314) or deficits of nitrate (*r*^2^ = 0.371, *p* = 0.047) or silicic acid (*p* = 0.659), probably because the shallow mixed layers (<15 m) do not mix phytoplankton deep enough to induce light limitation, making other factors more important in driving variability in phytoplankton growth.

Neither nitrate nor silicic acid flux are significantly correlated with their deficits or chlorophyll, showing that, in general, macronutrients were not limiting primary production during this study, consistent with previous findings at the WAP [30,39]. However, strong nitrate drawdown at station T05, where integrated chlorophyll was highest, highlights the potential for transient nutrient limitation in this setting. Iron concentrations were not measured, so we cannot assess directly their influence on primary production. However, Annett *et al.* [84] have shown that dissolved iron (dFe) concentrations decrease offshore and can become limiting over the shelf west of Marguerite Bay. Despite significant contributions (≥2.8%) of meteoric water, an important source of dFe at the WAP [84], to the upper 40 m at all stations (figure 2*d*), we cannot rule out a role for iron limitation in driving the observed variability in chlorophyll concentrations and nutrient deficits.

### (b) What causes nutrient enrichment across the shelf?

The increase in subsurface concentrations of macronutrients and inorganic carbon with cross-shelf modification of CDW strongly suggests that these physical changes are not primarily responsible for the nutrient enrichments. We hypothesize that these enrichments are driven by remineralization of organic matter and dissolution of biogenic silica as phytoplankton cells sink out of the surface ocean. We propose that remineralization by a microbial community including nitrifiers is most important for nitrogen, carbon and phosphorus and produces subsurface maxima in nitrate, phosphate and *p*CO_2_, which are enriched compared to the underlying CDW source. Brine rejection during sea ice formation may play a secondary role in enriching *p*CO_2_ and DIC in the Winter Water. We suggest that biogenic silica dissolves deeper in the water column, and that seafloor sediment porewaters may be a hotspot for dissolution, creating an important source of silicic acid to the water column. In the following, we use nutrient stoichiometric and isotopic data to support these hypotheses.

The cross-shelf reduction in N* in subsurface waters shows that, as nutrient concentrations are enriched across the shelf, phosphate is enriched to a greater degree than nitrate relative to the Redfield ratio on which N* is based. This is consistent with [NO_3_^−^]/[PO_4_^3−^] uptake ratios lower than 16 : 1 across much of the shelf, which produces organic matter with comparatively low N/P content and explains the observation of higher N* in surface waters at most stations. Subsequent remineralization of this low N/P organic matter acts to reduce N* in the subsurface. The rapid decrease in subsurface N* at the northern Marguerite Bay stations (T09, T10, CH1) is driven by a [NO_3_^−^]/[PO_4_^3−^] uptake ratio (13.1 ± 0.4) significantly lower than the Redfield ratio, thus particularly low N/P of organic matter. Low phytoplankton N/P is characteristic of blooming conditions [85,86] and diatom-dominated production [87–89], consistent with [Si(OH)_4_^−^]/[NO_3_^−^] uptake ratios ≥1 at the Marguerite Bay stations, where primary production is known to be diatom-dominated [35,36,90]. Subsurface N* is particularly low in Ryder Bay and the cold hole, because mCDW is blocked by bathymetric sills 350 and 150 m deep, respectively [61], such that a larger proportion of the nutrient pool is remineralized from low N/P organic matter and the proportion from mCDW is smaller compared to the other stations. Fine-scale minima in N* at the base of the euphotic layer at stations T03 and T06 may be driven by organic matter remineralization to phosphate and ammonium, rather than nitrate in the first instance. However, this is not likely to be significant in the deeper subsurface where remineralization to nitrate is expected to be complete. N* is relatively invariant below 200 m at each station, without consistently lower values in the deepest samples than in overlying mCDW, indicating that benthic denitrification does not play a significant role in lowering N* across the shelf.

This regeneration of nutrients and inorganic carbon by organic matter remineralization and subsequent nitrification can explain the cross-shelf increases in subsurface concentrations that we observe, as high-nutrient subsurface waters are enriched further by nutrients regenerated from sinking organic matter. Subsurface nitrite maxima, just below the surface fluorescence peaks and within the depth interval over which %OC and %N show marked declines with depth and *δ*^15^N_PN_ shows the strongest increases, provide good evidence for remineralization including nitrification, because nitrite is an intermediate product of the oxidation pathway. Below, we use the N and O isotopic composition of nitrate and *δ*^15^N_PN_ with complementary biogeochemical and physical data to explore these processes further and elucidate their importance for nutrient dynamics across the WAP shelf.

### (c) Nitrogen recycling and nitrification: evidence from nitrate and particulate nitrogen isotopes

The Rayleigh model describes the nitrogen isotopic enrichment of a pool of nitrate as it is used by phytoplankton in a closed system, due to the preferential uptake of the lighter energetically favoured ^14^N isotope. This model is considered most appropriate for nitrate consumption by phytoplankton blooms in the stratified Antarctic surface ocean [66,91]. The extent of kinetic fractionation is determined by the isotope effect or fractionation factor, *ε*, which is defined by equation (4.1), where ^14^*k* and ^15^*k* are the rate coefficients of the reaction for ^14^N- and ^15^N-containing nitrate:
4.1
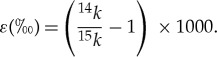

The *ε* value is low in the polar Southern Ocean as a result of a stratified upper water column and shallow mixed layers, where phytoplankton are alleviated from light limitation and its effect on *ε* [91]. Here we present model predictions based on 4‰ and 5‰, which is representative of the range found in this environment and consistent with *ε* calculated from 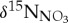 and *δ*^15^N_PN_ in this study (see below). The Rayleigh model is defined for 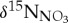 by equation (4.2), where 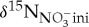 and [NO_3_^−^]_ini_ are the initial isotopic signature and concentration of the nitrate pool prior to uptake by phytoplankton, defined here as the mean of all values from the *T*_min_ layer which supplies nitrate to the surface ocean, 5.58 ± 0.07‰ (s.e., *n* = 24) and 29.77 ± 0.27 µmol l^−1^ (s.e., *n* = 24):
4.2
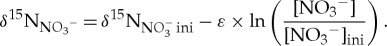


The same model can also be employed for 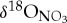, for which the initial value from the mean of all *T*_min_ values is 2.33 ± 0.08‰ (s.e., *n* = 20). The isotope effect of nitrate assimilation has been shown to be approximately equivalent for 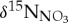 and 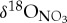 [92,93], such that good agreement of observed 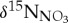 and 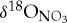 with the Rayleigh model would indicate that nitrate uptake by phytoplankton was the primary process acting on the nitrate pool. Deviation from the Rayleigh model and/or decoupling of 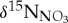 and 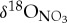 indicate that processes other than nitrate uptake are at work, in particular the recycling of nitrogen to resupply the nitrate pool. The 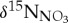 and 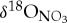 values and the difference between them (Δ15–18) are used here to identify these nitrogen cycle processes and the extent to which they influence the upper ocean nitrate pool in this region.

**Figure 8.**
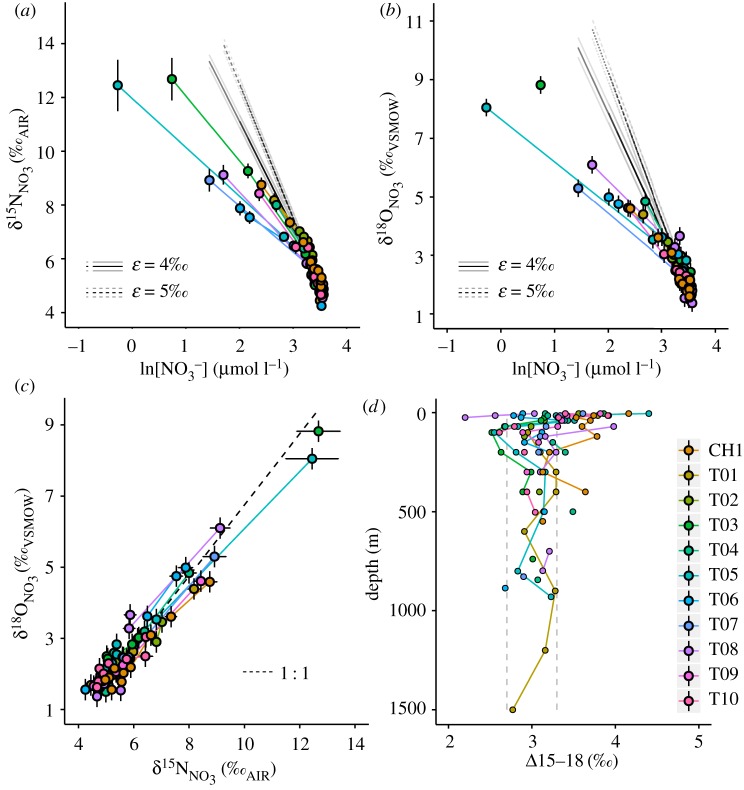
Plots of (*a*) 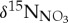 versus ln[NO_3_^−^], (*b*) 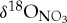 versus ln[NO_3_^−^] and (*c*) 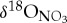 versus 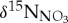, and (*d*) a depth profile plot of Δ15–18 for all stations, as per legend. In (*a*) and (*b*), black lines depict the modelled relationships using *ε* values of 4‰ (solid) and 5‰ (dashed); grey lines depict the standard errors of modelled values. In (*c*), the dashed line depicts a 1 : 1 enrichment ratio of 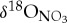 : 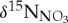. Error bars for 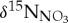 show the uncertainty associated with the correction for nitrite interference, which arises from the range of 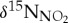 values measured in the Southern Ocean [64]. Error bars for 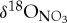 depict analytical error. In (*d*), dashed grey lines depict the expected range of Δ15–18 in CDW.

The 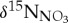 value falls below the modelled relationship based on nitrate uptake alone in surface water samples to varying degrees along the transect, with the largest data–model offsets at stations T05–T08 (figure 8*a*). 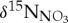 values estimated from measured 
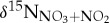
 by correcting for nitrite interference (see §2) are subject to uncertainty in the low *δ*^15^N of nitrite [64]. As a result of the low values of 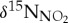, this correction increases 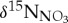 when compared with 
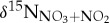
, but this increase is negligible (0.02 ± 0.03‰) below 70 m due to very low nitrite concentrations. In the upper ocean (≤70 m) where nitrite concentrations are higher, this correction increases 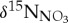 by 0.1–2.3‰, with the majority of values increasing by less than 0.6‰. Figure 8*a* shows that, even with the effect of low-*δ*^15^N nitrite removed and taking into account the uncertainty with this calculation, the deviation of our 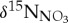 data from the Rayleigh model is robust.

The 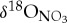 value shows a similar pattern, with most upper ocean data falling below the modelled relationship and the largest data–model offsets at T05–T07 (figure 8*b*). Data–model offsets for both 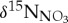 and 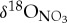 increase as nitrate is drawn down towards the surface. Apparent isotope effects of nitrate assimilation for the N (^15^*ε*_assim_) and O (^18^*ε*_assim_) isotopes calculated from the slopes of the regressions of 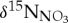 and 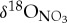 versus the natural logarithm of nitrate concentration (ln[NO_3_^−^]) are shown in table 2 for full-depth profiles and for mixed layer samples only. ^15^*ε*_assim_ based on full-depth profiles falls within the range of published values for the polar Antarctic zone at T01, T02 and T10 [62,91]. ^15^*ε*_assim_ calculated from 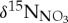 and *δ*^15^N_PN_ in this study is 4.7 ± 0.5‰ using the mean of instantaneous values (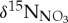 – *δ*^15^N_PN_) and 4.4 ± 0.4‰ assuming an accumulated product (equation (4.3)), in agreement with ^15^*ε*_assim_ based on 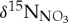 versus ln[NO_3_^−^] at stations T01 and T10:
4.3
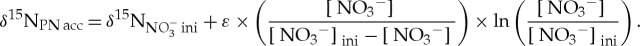

The ^15^*ε*_assim_ value is lower at most other stations, with statistically significant differences for stations T05–T08 and T03 compared to the value for T01 (*p* < 0.05, two-sample *t*-tests). ^18^*ε*_assim_ based on full-depth profiles is low at all stations, being similar to or lower than apparent ^15^*ε*_assim_ and minimal at stations T05–T07. Calculations of ^15^*ε*_assim_ and ^18^*ε*_assim_ based on mixed layer data only are within error of full-depth values for all stations. Isotope effects of nitrate assimilation of 1.5–3‰ can be ruled out for the summertime Southern Ocean [62,63,66], so the values that fall within this range are not the true isotope effects and are instead driven anomalously low by other processes.

**Table 2.** ^15^*ε*_assim_ and ^18^*ε*_assim_ estimated from the slopes of the relationships 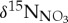 and 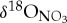 versus ln[NO_3_^−^] for each station using full-depth profiles and mixed layer data (mld) only. Values are only given for statistically significant relationships (*p* < 0.05), and uncertainties are standard errors. Sample number used for each regression is given in parentheses. NS denotes no significant relationship.

	^15^*ε*_assim_ full (‰)	^18^*ε*_assim_ full (‰)	^15^*ε*_assim_ mld (‰)	^18^*ε*_assim_ mld (‰)
T01	4.0 ± 0.4 (11)	3.0 ± 0.2 (10)	NS	NS
T02	6.0 ± 0.5 (9)	4.0 ± 0.8 (6)	NS	NS
T03	2.8 ± 0.1 (11)	2.4 ± 0.1 (9)	2.7 ± 0.2 (5)	2.3 ± 0.1 (4)
T04	3.6 ± 0.2 (10)	3.8 ± 0.3 (8)	3.6 ± 0.4 (4)	3.4 ± 0.3 (4)
T05	2.0 ± 0.1 (10)	1.6 ± 0.1 (10)	1.9 ± 0.1 (3)	NS
T06	2.0 ± 0.3 (11)	2.1 ± 0.2 (9)	1.2 ± 0.1 (4)	1.5 ± 0.1 (4)
T07	1.9 ± 0.1 (11)	1.6 ± 0.1 (7)	1.8 ± 0.1 (4)	1.6 ± 0.1 (3)
T08	2.3 ± 0.1 (10)	2.4 ± 0.4 (9)	2.0 ± 0.1 (3)	NS
T09	3.0 ± 0.2 (10)	2.3 ± 0.1 (7)	2.8 ± 0.04 (3)	NS
T10	4.2 ± 0.5 (11)	2.4 ± 0.4 (8)	NS	2.2 ± 0.2 (3)
CH1	3.3 ± 0.2 (10)	2.7 ± 0.2 (11)	3.0 ± 0.3 (4)	2.4 ± 0.5 (4)

In the open Southern Ocean, a lowering of both 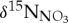 and 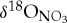 compared to the Rayleigh model, with a greater lowering of 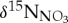 than of 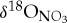, has been shown to indicate nitrification in the water column or in sea ice [53–55,65,94]. The lowering of 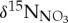 compared to the Rayleigh model is attributed to the nitrogen in nitrified nitrate coming from the remineralization of organic nitrogen with low *δ*^15^N (mean approximately 0‰), while 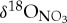 is lowered to a lesser extent because oxygen in nitrified nitrate is sourced from seawater oxygen and has a 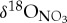 value approximately 1.1‰ higher than ambient seawater [95].

In this study of the WAP shelf environment, we find that 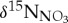 and 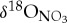 are both lowered compared to the Rayleigh model, but that 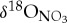 is lowered to a similar or greater extent than 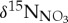 (figure 8*a–c*). We argue that these patterns are also driven by nitrification, with the lowering of 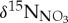 compared to the Rayleigh model driven by *δ*^15^N_PN_ being lower than 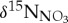 and the lowering of 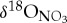 driven by incorporation of seawater *δ*^18^O lower than 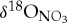. The key difference from the open Southern Ocean is greater nitrate drawdown in this high-productivity shelf setting, which leads to a higher 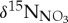 of nitrified nitrate. A greater degree of nitrate consumption by larger phytoplankton blooms and higher primary productivity results in significantly higher 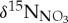 and consequently significantly higher *δ*^15^N_PN_ (greater than 3‰; figure 6*d*) than in the open Southern Ocean. According to the Rayleigh model, complete remineralization of an organic matter pool would produce regenerated nitrate with 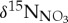 equal to *δ*^15^N_PN_. Complete remineralization is not likely in the upper ocean over the WAP shelf during summer, because estimates of organic matter export are up to approximately 50% of surface primary production [43–45], such that regenerated nitrate would have a lower 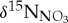 than if remineralization were complete. Nevertheless, the *δ*^15^N of nitrified nitrate is sensitive to the *δ*^15^N_PN_ of organic matter being remineralized, such that the high *δ*^15^N_PN_ that we observe in the high-productivity WAP shelf environment will produce nitrified nitrate with 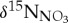 significantly higher than in the open Southern Ocean studies where *δ*^15^N_PN_ was much lower. As such, 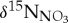 of nitrified nitrate over the shelf is lower than 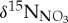 of the deep-sourced nitrate pool and causes a lowering of 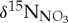 compared to the Rayleigh model, but to a lesser extent than in the open Southern Ocean where the difference between 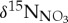 in nitrified and deep-sourced nitrate was much larger.

Seawater *δ*^18^O values in the upper ocean during this study were −0.71‰ to 0.17‰ (mean = −0.5 ± 0.1‰), consistent with observations across the WAP shelf during austral summers 2011–2014 [81]. With an enrichment of approximately 1.1‰, the 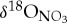 of nitrified nitrate is expected to be approximately 0.6‰ in this region, which is slightly lower than 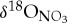 in the open Southern Ocean, due to the influence of low-*δ*^18^O glacial meltwaters from Antarctica. With 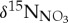 of nitrified nitrate being higher in this study than in the open Southern Ocean and 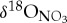 of nitrified nitrate being slightly lower, nitrification lowers both 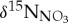 and 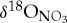 compared to the Rayleigh model, with 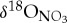 being lowered to a similar or greater extent than 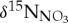. The difference in nitrate consumption and, therefore, 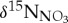 of nitrified nitrate between this and other Southern Ocean studies highlights a key difference in nitrate isotope systematics associated with nitrification between the open ocean and the high-productivity WAP shelf, which should be taken into account in future studies.

Nitrifiers are partly photo-inhibited, so low light levels are required for nitrification to proceed [96,97]. Summertime mixed layer nitrification over the Kerguelen Plateau has been attributed to phytoplankton dominating in the well-lit upper mixed layer and nitrifiers dominating in the low-light conditions deeper in the mixed layer [55]. Maximum nitrification rates are known to occur at the base of the euphotic layer, where PAR is 1–10% of its surface value [96]. Similar to Henley *et al.* [30], we use the depth at which PAR is 1–5% of its surface value to indicate the minimum depth where water column nitrification is likely to occur. These depth bands range from 10–16 m to 33–60 m across the WAP shelf, but lie between 10 and 18 m at the majority of stations, setting favourable conditions for nitrification in the upper ocean. Nitrification within and below these depth bands and entrainment of low-*δ*^15^N and low-*δ*^18^O nitrified nitrate into the surface layer cause the modification of isotopic signatures observed here, to varying degrees depending on the relative contribution of new versus regenerated nitrate. Nitrification in the mixed layer over winter is known to modify the isotopic signature of the Winter Water mass [53], but we do not observe anomalously low 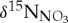 in the *T*_min_ layer, as a result of higher nitrate consumption, thus remineralization of organic matter with higher *δ*^15^N_PN_.

Intracellular enzyme-level interconversion between nitrate and nitrite has been demonstrated in the Pacific Antarctic surface ocean during autumn [64]. Kemeny *et al.* [64] suggested that this was driven by mixing of nitrifiers into the well-lit surface ocean through autumn mixed layer deepening, and photo-inhibition of nitrite oxidation, allowing the reversible nitrite oxidoreductase enzyme to catalyse nitrate–nitrite interconversion. While we are unable to assess directly the importance of this process here, because we did not measure *δ*^15^N and *δ*^18^O of both nitrate + nitrite and nitrate only, the observed increase in the difference between 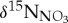 and 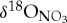 (Δ15–18; figure 8*d*) towards the surface is consistent with this mechanism, which would lower 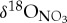 compared to 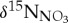. Further, the importance of subsurface nitrification that we have described and the shallow *T*_min_ depths that we observe are consistent with the presence of nitrifiers in the well-lit upper ocean, which could facilitate nitrate–nitrite interconversion. The fact that the 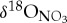 : 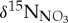 enrichment ratio is close to or only slightly less than 1 : 1 at the majority of stations (figure 8*c*) suggests that the effect of such interconversion is small compared to that of nitrification below the well-lit surface layer, but this mechanism is worthy of further study during austral summer.

Regardless of the degree of nitrate–nitrite interconversion, the subsurface nitrification that we have demonstrated can explain the progressive increase in nitrate concentration below the mixed layer as mCDW crosses the WAP shelf. This enrichment is driven by ongoing remineralization of sinking organic matter, as well as downward mixing of subsurface water masses rich in nitrified nitrate as the deepest, densest waters are progressively blocked [61].

### Other factors potentially influencing 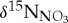 and 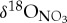


(d)

Nitrogen cycling processes in sea ice have been shown to influence 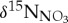 and 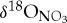 in the underlying seawater, through nitrification or sea ice algal nitrate uptake within the ice matrix [98]. However, we argue that neither of these processes can explain the lowering of 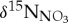 and 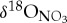 that we observe, primarily because sea ice was only present at station T07 and nitrate isotope dynamics were similar at this station to stations T05, T06 and T08 where the ice pack had retreated. Land-fast sea ice was present adjacent to Rothera Research Station in the weeks preceding the cruise [99] and is used here to consider the potential effect of sea ice processes on surface water biogeochemistry across the shelf. While sea ice nitrification in the region is supported by high concentrations of nitrite and ammonium in the sea ice at Rothera, its effect on surface water isotope signatures is limited by the small size of the sea ice nitrate pool. The maximum vertically integrated nitrate concentration in the sea ice at Rothera was 2.70 mmol m^−2^, which only accounts for 0.3% of the total nitrate pool in the biologically active upper ocean across the shelf (802 ± 123 mmol m^−2^). Using the lowest possible 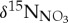 value for nitrified nitrate in the Southern Ocean, −13‰ [94], and assuming that all ice-derived nitrate was input immediately prior to sampling to yield the maximum possible effect on upper ocean nitrate, ice-derived nitrate would only lower upper ocean 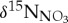 by a maximum of 0.06‰. As such, even if sea ice had been present during the cruise, nitrification within the ice matrix would not have had a significant effect on upper ocean isotope signatures, because of the small volume of low-nitrate sea ice meltwaters compared to the deep nutrient-rich ocean below. Similarly, we argue that sea ice is not a significant source of nitrate to the upper ocean across the WAP shelf, and, on the contrary, would act to dilute the upper ocean nitrate inventory [30].

Complete nitrate utilization in sea ice has the potential to draw down nitrate in the surface waters with no effect on 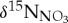 or 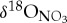, because no residual nitrate would be returned to the water column, which would lower the apparent isotope effect in surface waters. Even though sea ice was absent at all but one station during the cruise, algal production when sea ice was present prior to retreating southwards could have left a memory effect on surface water isotope signatures. However, we argue that this process did not exert a strong control on surface waters sampled during this study. Although nitrate assimilation is supported by dense accumulations of sea ice algae and an overall reduction in sea ice nitrate concentration between late November and late December close to Rothera, nitrate utilization was not complete [99]. Further, the ice at Rothera was relatively porous at the time of sampling, consistent with known sea ice conditions in Marguerite Bay and the adjacent shelf, even during winter [100]. As such, we suggest that exchange between the sea ice matrix and surface waters [78] would have prevented complete consumption of nitrate and limited its effect on surface ocean 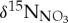 and 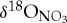.

A contribution of Lower CDW to the surface nitrate pool, as observed south of Australia [91], and the inappropriateness of the Rayleigh model due to sporadic wind-driven mixing events resupplying nitrate from depth, also have the potential to modify upper ocean 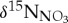 and 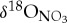. However, neither of these hydrographic explanations can account for the lowering of 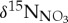 and 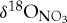 here, because the difference in isotopic signatures between Upper and Lower CDW is too small, and nutrient resupply driven by vertical mixing would increase [NO_3_^−^] while lowering 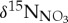 and 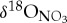, moving data points along the modelled line towards initial values, rather than causing the observed data–model deviation. Furthermore, the well-stratified upper ocean conditions across the WAP shelf during the study probably restricted mixing events and nutrient resupply, in keeping with the Rayleigh model.

### (e) Importance of nitrification to the upper ocean nitrate inventory

While the ubiquity of nitrification in the subsurface ocean is well established, the influence of nitrification in the Southern Ocean surface layer during winter and at the base of the euphotic zone during summer on upper ocean nitrate isotope systematics is increasingly being recognized [30,53–55]. High nutrient utilization in surface waters over the high-productivity WAP shelf allows us to use nitrate isotopic signatures to estimate the local contribution of nitrate regenerated by organic matter remineralization and nitrification to the upper ocean nitrate pool, as opposed to that supplied from CDW. To do this, we perform an isotopic mass balance calculation for stations along the transect according to equation (4.4), where 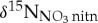 is the value of nitrified nitrate, for which we use two endmember values applicable to Southern Ocean conditions, −3.5‰ to −13‰ [94]:
4.4
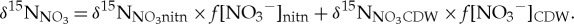

The 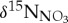
_CDW_ value is a modelled value based on uptake of CDW-sourced new nitrate with an isotope effect of 4‰ (equation (4.2)). *f*[NO_3_^−^]_nitn_ and *f*[NO_3_^−^]_CDW_ are the estimated fractions of total nitrate from nitrification and CDW, respectively, and are presented with input data in table 3. These calculations were performed for each value of 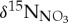
_nitn_ for each station where the difference between 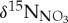
_CDW_ and 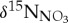 is statistically significant (*p* < 0.05, two-sample *t*-tests). Based on the uppermost sample at each station, where nitrate drawdown was greatest, so that the isotopic effect of nitrate regeneration was expressed most clearly (data–model 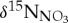 offsets were greatest), the contribution of regenerated nitrate to the total upper ocean nitrate pool was up to 32% based on 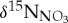
_nitn_ of −3.5‰ and up to 23% based on 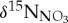
_nitn_ of −13‰. These estimates are in broad agreement with estimates of the contribution of regenerated phosphate to the total phosphate pool (table 3), calculated from apparent oxygen utilization (AOU) following Ito & Follows [101].

**Table 3.** Input parameters and outputs from equation (4.4) for each station, as well as estimates of regenerated phosphate calculated from (AOU/150)/[PO_4_^3−^] [101,102]. % [NO_3_^−^]_nitn_ was calculated only where the difference between 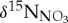
_CDW_ and 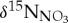 was statistically significant (*p* < 0.05, two-sample *t*-tests). NS denotes differences not statistically significant. Uncertainties represent standard errors.

	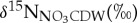	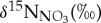	% [NO_3_^−^]_nitn_ ( 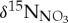 _nitn_ = −3.5 ‰)	% [NO_3_^−^]_nitn_ ( 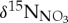 _nitn_ = −13 ‰)	P_reg_ % (AOU)
T01	8.5 ± 0.2	8.2 ± 0.2	NS	NS	16 ± 1
T02	6.7 ± 0.2	7.0 ± 0.2	NS	NS	15 ± 1
T03	16.1 ± 0.3	12.7 ± 0.2	17 ± 0.4	12 ± 0.3	16 ± 2
T04	8.4 ± 0.2	8.0 ± 0.2	NS	NS	11 ± 1
T05	20.1 ± 0.4	12.5 ± 0.2	32 ± 0.8	23 ± 0.6	21 ± 2
T06	11.1 ± 0.2	7.9 ± 0.2	22 ± 0.8	13 ± 0.4	18 ± 2
T07	13.3 ± 0.2	8.9 ± 0.2	26 ± 0.8	17 ± 0.5	33 ± 2
T08	12.3 ± 0.2	9.1 ± 0.2	20 ± 0.6	13 ± 0.4	26 ± 2
T09	9.6 ± 0.2	8.4 ± 0.2	9 ± 0.3	5 ± 0.2	30 ± 2
T10	6.9 ± 0.2	6.4 ± 0.2	NS	NS	29 ± 2
CH1	9.5 ± 0.2	8.8 ± 0.2	NS	NS	21 ± 2

Regenerated phosphate was lower over the outer shelf, with a minimum value of 11 ± 1% of total phosphate at station T04, and higher at stations within Marguerite Bay, with a maximum value of 33 ± 2% at station T07. Regenerated phosphate values are consistent with nutrient regeneration occurring ubiquitously in the subsurface. The extent to which this regeneration is expressed in nitrate isotope signatures depends on the degree of nitrate utilization, such that the lowering of both 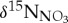 and 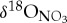, and consequently the estimated contribution of regenerated nitrate, is greatest at stations T05–T08 and T03 where nitrate utilization is greatest. Estimates of nutrient regeneration from regenerated phosphate contribution and 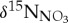 are in agreement for stations T03 and T06, suggesting that both estimations give realistic values for nutrient regeneration. In cases where the regenerated phosphate contribution is greater than the value for nitrification from 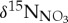, we suggest that the phosphate-derived value is more realistic and the nitrification value is an underestimate due to underexpression of the isotopic effect of nitrate regeneration. Station T05 is the only station for which the regenerated phosphate contribution falls at the lower end of values for nitrification, which could potentially indicate that 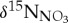 is lowered further by another factor in addition to nitrate regeneration. As surface layer nitrate concentration at this station was 0.76 µmol l^−1^ at the time of sampling, transient nutrient limitation could have led to underexpression of the organism-level isotope effect and thus a contribution to low 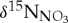 compared to the Rayleigh model. Such low nitrate concentrations were not observed at any other station.

Broad agreement between the contribution of regenerated nutrients to the total upper ocean nutrient pool derived from regenerated phosphate and nitrate isotope signatures suggests that up to one-third of the phosphate and nitrate in the WAP surface ocean is regenerated locally, rather than being supplied from CDW. This has implications for our understanding of new production versus regenerated production if up to one-third of the nitrate and phosphate pools are themselves regenerated, and could lower our estimates of net biological carbon uptake based on nutrient uptake by the same proportion. The fact that the regenerated phosphate contribution is significantly higher at the inner-shelf stations (T07–T10) than mid- and outer-shelf stations (T01–T06) (*p* = 3.75 × 10^−11^, two-sample *t*-test) further supports the argument that biological nutrient regeneration can explain the progressive enrichment in subsurface nutrient concentrations across the shelf.

### (f) Macronutrient and inorganic carbon enrichment across the shelf

We have used nutrient and organic matter concentrations and nitrate and PN isotopes to show that remineralization of organic matter sinking out of the surface ocean and subsequent nitrification in the subsurface, where light levels are sufficiently low, can explain the observed increase in nitrate concentration below the surface layer across the WAP shelf. Similar enrichment in phosphate across the shelf and the strong positive linear correlation between [NO_3_^−^] and [PO_4_^3−^] (*r*^2^ = 0.983, *p* < 2.2 × 10^−16^; figure 7*a*) suggest that organic matter remineralization is also the primary driver of cross-shelf enrichments in phosphate. This is supported further by broad agreement between estimations of local biological nutrient regeneration based on regenerated phosphate and nitrate isotopes.

Nitrate and DIC show a strong positive correlation from the surface to the Winter Water layer (*r*^2^ = 0.969, *p* < 2.2 × 10^−16^, *n* = 62; figure 7*d*), yet deviation from this relationship below the Winter Water layer shows that DIC was enriched in the warm CDW compared to nitrate relative to their relationship in the upper ocean. Alongside depth profile plots that show a shallower subsurface nitrate maximum than for DIC, we interpret these relationships as showing extensive regeneration of nitrate and phosphate above and within the Winter Water layer, with regeneration of DIC continuing into the mid-depths, creating a deeper maximum below 200 m. These relationships agree well with organic nitrogen being more labile than organic carbon, thus being recycled earlier during export.

Brine rejection can act to enrich DIC and *p*CO_2_ in the Winter Water layer [47], where a negative sea ice meltwater fraction indicates net sea ice formation during the previous winter, particularly in Marguerite Bay (*T*_min_ to 100–200 m; figure 2*e*). While strong increases in *p*CO_2_ and DIC over this depth interval (figure 5) may indicate a role for brine rejection, we argue that subsurface maxima of *p*CO_2_ and DIC below the Winter Water show that remineralization of sinking organic matter from the spring/summer phytoplankton bloom is more important in driving the cross-shelf enrichments.

The continual increase of silicic acid with depth, and the cross-shelf increases in silicic acid and Si* occurring deeper than those of nitrate, phosphate and inorganic carbon (figure 3), are consistent with remineralization of biogenic silica occurring deeper than that of organic matter [103] and suggest that this is not complete before reaching the seabed in this region. Silicic acid hotspots observed near the sediment–water interface, particularly with departures from nitrate shown by high Si*, may also suggest a significant sedimentary silicic acid source to the water column, with its effect on bottom water biogeochemistry dependent on residence time. Arctic shelf sediments can be a significant source of silicic acid to bottom waters, due to dissolution of biogenic silica from the overlying water column in sediment porewaters [104]. We hypothesize that a similar sedimentary source may be important in enriching silicic acid across the WAP shelf, and arises from dissolution of diatom-derived biogenic silica in shelf sediment porewaters and flux of silicic acid back to the water column. We speculate that such benthic fluxes may also be important elsewhere around Antarctica, and this should be a priority for future research.

## 5. Conclusion

The processes that regulate nutrient supply and cycling are of fundamental importance to the functioning of the productive WAP shelf ecosystem. We show enrichments of nitrate, phosphate, inorganic carbon and silicic acid in subsurface waters as warm, nutrient- and carbon-rich CDW crosses the WAP shelf. This is contrary to cross-shelf trends in physical parameters, which show loss of heat, salt and density, such that physical modification of CDW is not the primary driver of cross-shelf changes in nutrient biogeochemistry. While fluxes of these nutrients into the upper ocean did not limit primary production overall during this study, transient nutrient limitation can occur when chlorophyll is high and the mixed layer is shallow.

We present nutrient stoichiometric and isotopic evidence for remineralization of sinking organic matter and significant nitrification below the euphotic surface layer across the WAP shelf during summer, and argue that this is the primary driver of the observed cross-shelf nutrient enrichments in subsurface waters. Both *δ*^15^N and *δ*^18^O of nitrate in the upper ocean are lowered compared to values expected for nitrate utilization alone, and we attribute this to the effect of subsurface nitrification on the surface nitrate pool. A key difference between the high-productivity WAP shelf and the open Southern Ocean is the higher degree of nitrate consumption over the shelf and, therefore, the higher *δ*^15^N of nitrified nitrate, which holds important consequences for the nitrate isotope systematics associated with nitrification. Greater nitrate consumption in the shelf environment also allows us to use 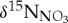 to estimate the proportion of the surface nitrate pool that is regenerated locally compared to that supplied from CDW. Broad agreement between these estimates and estimates of regenerated phosphate from AOU suggests that locally regenerated nitrate and phosphate can account for up to one-third of the surface nutrient pools, with the extent to which this is expressed in nitrate isotope signatures dependent on the degree of nitrate drawdown. These significant contributions of locally regenerated nutrients hold potentially important implications for net biological uptake of CO_2_ and the seasonal carbon sink. Regenerated phosphate contribution is significantly higher at the inner-shelf stations, further supporting the argument that biological nutrient regeneration can explain the progressive enrichment in subsurface nutrient concentrations across the shelf, particularly in the inner-shelf regions.

Similar patterns in nitrate, phosphate, DIC and *p*CO_2_ with depth and across the shelf, and relationships between nitrate and DIC, strongly suggest that cross-shelf enrichments of inorganic carbon are also driven primarily by remineralization of organic matter, with remineralization persisting into the mid-depths. Silicic acid concentrations are enriched by dissolution of biogenic silica, which occurs deeper in the water column than organic matter remineralization and is not complete before reaching the seafloor. Strong enrichments close to the sediment–water interface lead us to propose a potentially significant sedimentary source of silicic acid to WAP shelf bottom waters, most likely driven by dissolution of biogenic silica in sediment porewaters, with implications for benthic–pelagic coupling in this region.

## Data accessibility

All data are freely available from the British Oceanographic Data Centre (BODC) at www.bodc.ac.uk/data/published_data_library/catalogue. DOI: doi:10.5285/65576513-1638-2c67-e053-6c86abc09597. Short DOI: doi:10/ckn8 [105].
